# Exploring deep learning methods for recognizing rare diseases and their clinical manifestations from texts

**DOI:** 10.1186/s12859-022-04810-y

**Published:** 2022-07-06

**Authors:** Isabel Segura-Bedmar, David Camino-Perdones, Sara Guerrero-Aspizua

**Affiliations:** 1 Human Language and Accesibility Technologies, Computer Science Department, Universidad Carlos III de Madrid, Avenidad de la Universidad, 30, Leganés, 28911 Madrid, Spain; 2 Tissue Engineering and Regenerative Medicine group, Department of Bioengineering, Universidad Carlos III de Madrid, Avenidad de la Universidad, 30, Leganés, 28911 Madrid, Spain; 3 Hospital Fundación Jiménez Díaz e Instituto de Investigación, FJD, Av. de los Reyes Católicos, 2, 28040 Madrid, Spain; 4 Epithelial Biomedicine Division, CIEMAT, Avda. Complutense 40, 28029 Madrid, Spain; 5 Centre for Biomedical Network Research on Rare Diseases (CIBERER), C/Monforte de Lemos 3-5, 28029 Madrid, Spain

**Keywords:** Rare diseases, Named entity recognition, Deep learning

## Abstract

**Background and objective:**

Although rare diseases are characterized by low prevalence, approximately 400 million people are affected by a rare disease. The early and accurate diagnosis of these conditions is a major challenge for general practitioners, who do not have enough knowledge to identify them. In addition to this, rare diseases usually show a wide variety of manifestations, which might make the diagnosis even more difficult. A delayed diagnosis can negatively affect the patient’s life. Therefore, there is an urgent need to increase the scientific and medical knowledge about rare diseases. Natural Language Processing (NLP) and Deep Learning can help to extract relevant information about rare diseases to facilitate their diagnosis and treatments.

**Methods:**

The paper explores several deep learning techniques such as Bidirectional Long Short Term Memory (BiLSTM) networks or deep contextualized word representations based on Bidirectional Encoder Representations from Transformers (BERT) to recognize rare diseases and their clinical manifestations (signs and symptoms).

**Results:**

BioBERT, a domain-specific language representation based on BERT and trained on biomedical corpora, obtains the best results with an F1 of 85.2% for rare diseases. Since many signs are usually described by complex noun phrases that involve the use of use of overlapped, nested and discontinuous entities, the model provides lower results with an F1 of 57.2%.

**Conclusions:**

While our results are promising, there is still much room for improvement, especially with respect to the identification of clinical manifestations (signs and symptoms).

## Introduction

Rare diseases are characterized by a low prevalence in the population. There is no consensus on the percentage of affected people with a disease to be considered as a rare disease. Thus, whereas in the United States, a rare disease affects fewer than 200,000 people, in Europe, the prevalence of a rare disease is less than 1 person per 2000 [1]. To date, there are around 7000 rare diseases and new rare diseases are identified each week. In spite of their low prevalence, these diseases may affect more than 400 million people around the world [2, 3].

**Fig. 1:**
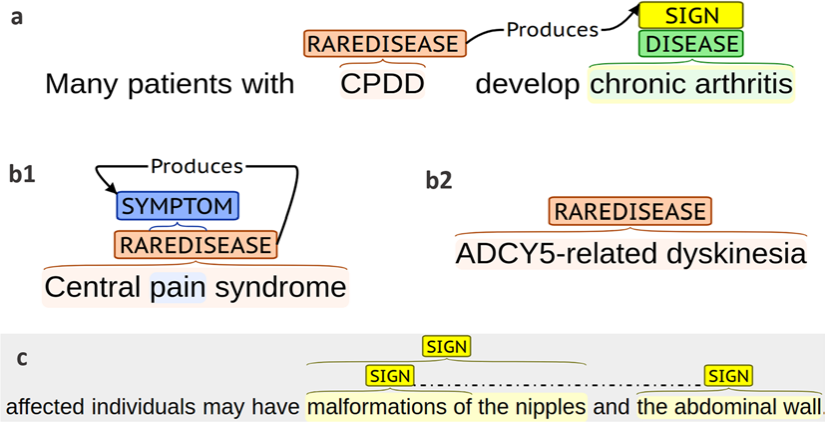
This figure shows some annotated sentences in the RareDis corpus. Sentence (**a**) shows an example of two overlapping entities: sign and diseases. It also has a 'Produces' relationships between a rare diseases and a sign. Sentence (**b1**) contains an example of nested name entities belonging to different entity types: symptom and rare disease. **b2** is a mention of rare diseases, which is muti-token. Sentence **c** contains several discontinuous mentions of signs

The diagnostic process of rare diseases becomes a very long road for patients and their families to obtain an accurate diagnosis and then receive an adequate treatment. The delay in diagnosis of rare diseases is between six and seven years [4]. A possible cause of the delayed diagnosis is the limited experience and knowledge about rare diseases of clinicians [5–7]. In addition, rare diseases may present a heterogeneous phenotype, with a wide variety of symptoms and signs, related among others with different driving mutations [8]. Both signs and symptoms are clinical manifestations of diseases [9]. A sign is an objective evidence, for example “malformation of the nipples” (see Fig. 1c), while a symptom is a subjective experience that can only be identified by the patient, for example “pain” (see Fig. 1b1). Since a rare disease can be associated with very different clinical manifestations [10], this fact can make early and accurate diagnosis enormously difficult. Therefore, there is an urgent need to increase the usability of the sparse and fragmented scientific and medical knowledge about rare diseases [11].

Artificial Intelligence, and in particular Natural Language Processing (NLP) and Machine Learning, can play a beneficial role by providing better access to the relevant information about rare diseases and their clinical manifestations (signs and symptoms), and in this way, helping to alleviate the workload on doctors. Although much of the knowledge about rare diseases is stored in databases and ontologies, biomedical literature (research articles, clinical cases, health forums, social media, etc) is a rich source of information about rare diseases in unstructured text. Information extraction techniques such as Named Entity Recognition (NER) can help structure this information, facilitating access to the knowledge embedded within those texts and boosting scientific research.

The automatic recognition of disease named entities has attracted much attention over the last years [12–18], as it can be applied in meaningful clinical applications such as cohort selection for clinical trials or epidemiological studies, pharmacovigilance, personalized medicine, among many others. This task is a very challenging task due to the diversity and complexity of disease names. Many disease names can have different synonyms and abbreviations to represent them. For instance, “obsessive-compulsive disorder”, “obsessive compulsive disorder”, “anancastic neurosis”, and “OCD” are the same disease. Moreover, disease names usually contain modifiers that can be related to body parts or degrees of disease (e.g., “periodic limb movement disorder” or “advanced sleep phase syndrome”). The recognition of symptoms and signs also presents additional challenges. Many symptoms and signs can be described by technical terms (e.g., “dysuria”), but also by short phrases (such as “pain or discomfort when you urinate”). Furthermore, other NER challenges such as overlapping, nested and discontinuous entities have received limited attention [19].

The recent advancements of deep learning models have facilitated great progress in NLP. Recently, transformers [20] and Bidirectional Encoder Representations from Transformers [21] have outperformed traditional and deep learning models for most of NLP applications [22–25], and in particular, for NER in the biomedical domain [17, 26].

We briefly describe the most recent deep learning approaches for recognizing diseases in biomedical texts. One of the first studies that applied deep learning to this task is described in [12]. The authors proposed a hybrid system composed of two modules: a Conditional Random Field (CRF) [27] trained with orthographic, morphological, and domain features from Unified Medical Language System (UMLS) [28], and a bidirectional recurrent neural network (RNN) initialized with domain-specific word embeddings. Finally, a Support Vector Machine (SVM) classifier is used to combine the outputs of the two previous modules. For the training and testing of the system, the authors used the dataset of the Disease Named Entity Recognition and Normalization (DNER) shared task  [29] of the BioCreative V challenge, which consists of 1500 PubMed abstracts and a total of 12,850 disease mentions. CRF achieves better results (F1=82.88%) than the bidirectional RNN (F1=78.27%). The output fusion by SVM obtains the best performance with an F1 of 84.28%.

In the last years, Bidirectional Long Short Term Memory (BiLSTM) [30] with CRF has proved to be the most successful model for the task of biomedical NER  [13, 31, 32]. The approach proposed by Habibi et al. [13] was one of the first works to exploit pre-trained word embeddings to initialize a BiLSTM+CRF network for recognizing diseases. The authors used two pre-trained embedding models created by Pyysalo et al. [33]. The first model (from now on called PubMed-PMC) was trained using a collection of texts formed by all abstracts from PubMed (more than 23 million abstracts) and all full articles from PMC (a database of open access with more than 700,000 full articles from the biomedical domain). The second embedding model (from now on called Wiki-PubMed-PMC) was an extension of the first one by adding approximately four million English articles from Wikipedia. These models were trained using the word2vec tool  [34]. The authors also trained a word embedding model by using a collection of 20,000 European patents. To train and evaluate their models, they use the NCBI corpus [35] and the CDR corpus [36]). The NCBI corpus is a collection of 793 PubMed abstracts and contains a total of 6892 disease mentions. The CDR corpus contains 1500 MEDLINE abstracts annotated with 5818 diseases, 4409 chemicals, and 3116 chemical-disease interactions. The experiments showed that the network initialized with Wiki-PubMed-PMC obtains better performance (with an F1 of 90.4% over the NCBI dataset and 88.17% over the CDR dataset) than those initialized with the other pre-trained models. This may be because the Wiki-PubMed-PMC model was trained on a larger collection of texts than the other pre-trained models. Moreover, this collection contained domain-specific and nonspecific texts.

The SBLC model [14], is also based on a BiLSTM network with a CRF layer. To represent the text, the authors trained a word embedding model by using a large collection of texts collected from PubMed, PMC, and Wikipedia, with a total of 5.5 billion words. The SBLC was trained and tested on the NCBI dataset, obtaining an F1 of 86.2%.

Instead of using RNN, Zhao et al.  [15] used a deep convolutional neural network (CNN). In addition to word embeddings, the authors also exploited character embeddings and lexicon feature embeddings to represent the texts. The character embeddings were generated by using a CNN layer. The MEDIC vocabulary [37], composed of more than 67,000 disease mentions, was used to create the lexicon feature embeddings. After the embedding layer, where each word is represented by concatenating its three embeddings, several CNN layers are applied to obtain higher level features. Then, instead of a CRF classifier, a multiple label strategy (MLS) is applied to capture the labels of the context words. This strategy uses a softmax function to obtain the probability of each possible label. The system obtained an F1 of 85.17% on the NCBI corpus, and an F1 of 87.83% on the CDR corpus.

Ling et al. [16] also used an architecture composed of a BiLSTM with a CRF layer. This architecture was initialized by using the three type of embeddings proposed by Zhao et al.   [15], as just described above. The main difference is that these authors applied a combination of a CNN and a LSTM to generate the character embeddings, instead of using a CNN network. The final model achieved an F1 of 83.8% on the NCBI dataset.

One of the main drawbacks of the pre-trained word embeddings models is that they only provide a vector for each word, so they do not handle polysemous words. Recently, contextualized word representation models (such as ELMo [38], GPT-2 [39] or BERT [21]) have emerged as an alternative to the non-contextual word embedding models, providing a different vector for each sense of a word. Lee and colleagues [17] applied BERT to the task of disease recognition on the NCBI dataset, achieving an F1 of 88.60%. The authors also trained their language representation model (BioBERT) on two large biomedical corpora such as PubMed and PMC. BioBERT slightly overcomes BERT on the NCBI dataset, with an improvement of 0.62%.

Li et al. [18] also trained a BERT model using 1.5 million electronic health record notes. This model was evaluated on the NCBI and CDR datasets, showing an F1 of 89.92% and 93.82% respectively.

Very few research efforts have focused on the extraction of rare diseases. The RDD corpus [40] contains 1000 MedLine abstracts covering 578 rare diseases and 3678 annotations expressing a disability. The authors analyzed a model based on Bi-LSTM and CRF to extract rare diseases and disabilities, achieving an F1 of 70.1% for rare diseases and 81% for disabilities.

In this paper, we address the task of recognizing rare diseases as well as their clinical manifestations (symptoms and signs). Moreover, to the best of our knowledge, this is the first work that explores three BERT-based models to extract rare diseases from texts. In particular, we use the basic BERT model and two models, BioBERT [17], and ClinicalBERT [41], which were trained using biomedical and clinical texts, respectively. In order to provide a comprehensive comparison, we also study several BiLSTM models initialized with different pre-trained word embedding models.

## Methods

### Dataset

We use the RareDis corpus [42], which is a collection of texts from the Rare Disease database (NORD)^1^. These texts were manually annotated with four entity types (diseases, rare diseases, signs, and symptoms). The corpus also includes relations between entities, but they are outside the scope of this work. The corpus has three different splits: training set, validation set, and test set. Table 1 shows the number of the entity types annotated, as well as the number of documents, sentences, and tokens in each split. A more detailed description of the RareDis corpus can be found in [42]. The corpus contains a total of 9318 entities. We can observe that sign and rare disease entity types are the most prevalent, around 41% and 34%, respectively. The disease entity type is the third-largest type, with approximately 17%, while symptom entity type is the most sparse entity type in the three splits.

**Table 1:** Statistics of the RareDis corpus

	Training	Validation	Test	Total
Documents	729	104	208	1041
Sentences	6451	903	1787	9141
Tokens	135,656	18,492	37,893	192,041
Diseases	1647	230	454	2331
Rare Diseases	3608	525	1095	5228
Symptoms	319	24	54	397
Signs	3744	528	958	5230

The corpus is distributed in Brat standoff format [43]. The RareDis corpus and its guidelines are publicly available for the research community^2^.

### Approaches

**Fig. 2:**
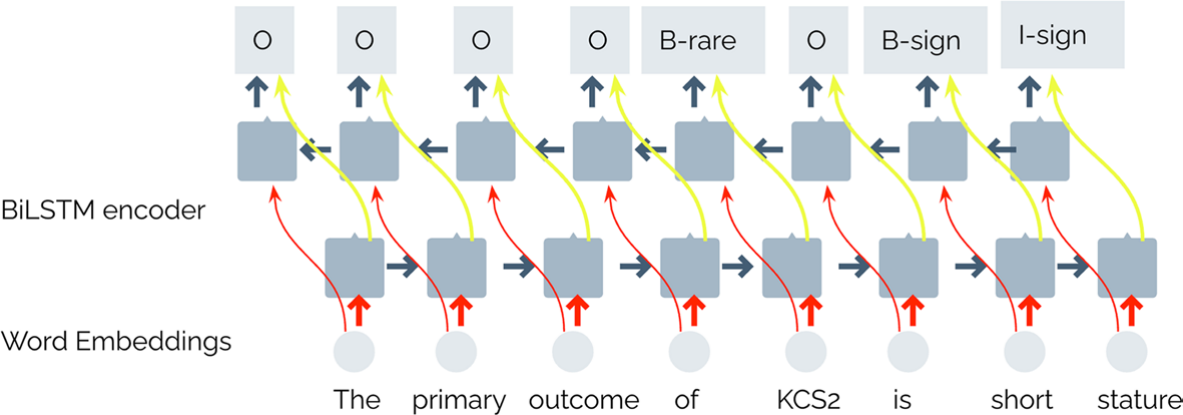
BiLSTM method. This figure shows the architecture of the BiLSTM network followed by a softmax layer

NER is a sequence labeling problem, where the goal of the model is to classify each token of the input sequence into the corresponding category. To define these categories, we must consider the types of entity that we intend to extract. As many entity mentions are multi-token, that is, they are composed of several words, for example, “ACDY5-related dyskinesia” (see Fig.  1), we must use a format that allows us to represent if a token belongs or not to a entity mention. Moreover, if the token does belong to an entity mention, we are interested in knowing if the token appears at the beginning of the mention or if it is an internal one of it. Typically, sequence labeling tasks use some of the variations of the format IOB encoding scheme [44], to represent the tokens. In our case, we represent each token using the standard IOB2 (Inside, Outside, Beginning) format [45], where B-X identifies the first token of an entity mention whose type is X (for example, B-SIGN), I-X identifies the continuation of an entity mention with type X (for example, I-SIGN), and O for other tokens. In this regard, the following nine categories or labels are used: O, B-Disease, I-Disease, B-RareDisease, I-RareDisease, B-Sign, I-Sign, B-Symptom, and I-Symptom. Thus, each of our proposed models should address this NER task as a multi-class classification problem and should produce one of these labels for each token in the input sequence. Figure 2 shows an example where an input sequence is processed by a BiLSTM network, where the last layer produces a label for each token in the input sequence. In this sequence, some tokens such as “outcome” or “primary” were classified with the label ’O’ (no entities). We can also see that “KCS2”, which is a rare disease mention formed by just one token, should be classified with the label B-RareDisease (we used the short label B-rare in the figure), while the following token, “is” should be labeled with “O”. This figure also provides an example of multi-token entity, “short stature”. The model should classify its first token as “B-sign”, indicating that this token is at the beginning of the mention, while the second one with ’I-sign’, indicating the token is inside of the mention.

Now, we describe the different methods used to deal with the task of NER on the RareDise corpus.

#### Conditional random fields (CRF)

As a baseline method, Conditional Random Fields (CRF) [27] is proposed. This is one of the most successful algorithms for any sequence labeling task such as NER [46, 47]. CRF learns the correlations between labels and provides the output sequence of IOB tags with the highest probability. That is, CRF predicts the most likely IOB tag for each token in the input sequence.

To represent each token, we consider three kinds of features: token, lemma, and PoS tag. We use Spacy [48], a very popular NLP library, to parse each input sequence and to obtain these features. For each token, we also select a window of size two. Then, the features (token, lemma and Pos tag) of the tokens belonging to this window form the feature set to represent each token. These features are fed into the CRF classifier, which predicts an IOB tag for each input token. To implement the model, we use the CRFSuite package [49]. The classifier was trained using both training and validation datasets since we use default hyperparameters. The Limited Memory Algorithm for Bound Constrained Optimization (L-BFGS) is used as the optimization method.

#### Bidirectional long short-term memory (BiLSTM)

BiLSTM has been successfully applied to the NER task in the biomedical domain [31, 50], and in particular, to recognize disease names [12–14, 16]. This model consists of a forward LSTM (which sequentially processes the input sequence from left to right) and a backward LSTM (which processes the input sequences from right to left). In this way, BiLSTM can learn relevant information from the previous and next context for each input token, effectively increasing the amount of information available to the network [51].

Our architecture consists of several layers (see Fig.  2), which are described below. First, in the input layer, the text is represented as word vectors. Then, these input vectors are passed to the BiLSTM layer described above. The output vector of the BiLSTM layer is the concatenation of the forward LSTM and the backward LSTM. After the BiLSTM layer, we consider two different strategies for the output layer.

The first strategy (see Fig. 2) is using a time-distributed dense (TDD) layer to classify each token by determining its most likely label. This layer applies a dense layer on each times-tep of the the BiLSTM network. In this layer, each conditional probability is assessed independently of the other conditional probabilities.

**Fig. 3:**
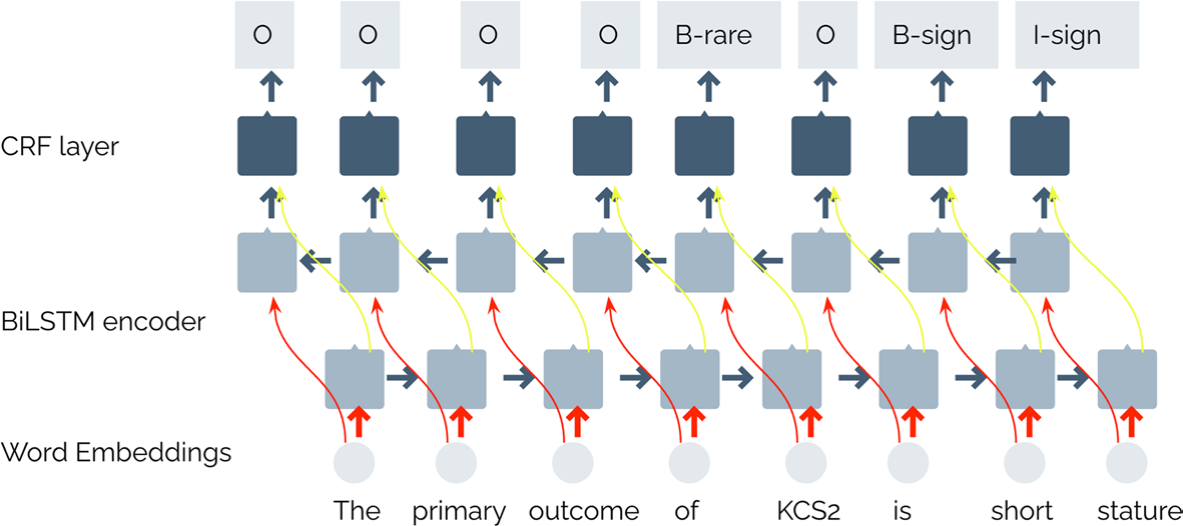
BiLSTM + CRF method. This figure shows the architecture of the BiLSTM network with a CRF classifier

The second strategy is using a CRF classifier as the last layer (see Fig. 3), which will output the sequence of IOB tags with the maximum probability for the input sequence. The CRF layer takes as input the label probability for each word coming from the output layer of the BiLSTM network. Thus, the context surrounding the label assignment predicted by the BiLSTM model is also added, whereby linear-chain CRF explicitly models dependencies between the labels through a transition matrix with transition scores between all pairs of the labels. This allows to easily learn constraints such as, for example, “I-RAREDISEASE” tag cannot follow an “O” tag. These types of constraints are captured by the CRF layer in a simple way by considering the time step in each token.

Moreover, we explore the effect of input text representation on the performance of BiLSTM. Texts must be encoded as vectors of real numbers to be used as input for machine learning and deep learning models. In the case of neural networks, it is possible to create a random vector for each input token. During the training, the network will adjust these word vectors alongside the other weights of the network. An alternative way is to represent tokens with word vectors (word embeddings) from a pre-trained language model. In the last decade, neural network language models [52, 53] have effectively replaced traditional models such as the Bag-Of-Words, achieving state-of-the-art results in many NLP tasks. Several studies have shown that word embeddings trained with neural networks can capture semantic and syntactic between tokens [34], providing thus an accurate meaning representation of the input tokens. The most popular word embeddings models are Word2Vec [34], Glove [54] and fastText [55]. In this work, we study the effect of different pre-trained word embeddings on the BiLSTM performance. In particular, we explore three different models:

- GoogleNews [56], a pre-trained word embedding model trained with the Word2Vec network on the GoogleNews dataset. The model contains word embeddings of dimension 300 for 3 million words.
- GloVe [54], a pre-trained word embedding model trained using Common Crawl, an open repository of web crawl data. The model contains 300-dimensional vectors for 840 billion tokens.
- PubMed, PubMed Central, and Wikipedia (Wiki-Pubmed-PMC) [57], a pre-trained word embedding model trained with the Word2Vec network on a collection of more than 23 million abstracts from PubMed (a database containing abstracts of scientific articles from the biomedical domain), 700,000 articles from PMC and around four million English Wikipedia articles. The dimension of the word embeddings is 200.

To implement and train the BiLSTM models, we use the Keras Python API [58] with TensorFlow as the backend. We use an Adam optimizer [59] with a learning rate 0.001 and categorical cross-entropy as a loss function. To avoid overfitting, we use early stopping with the patience of four, meaning that training will finish if the loss function does not improve in four consecutive epochs.

#### Bidirectional encoder representations from transformers (BERT)

Deep contextualized language models are capable to capture word meanings and their more representative relations with other words. Thanks to this accurate linguistic representation, these models achieved unprecedented results on many NLP tasks [21]. Moreover, contextualized language models are trained through unsupervised learning, requiring only a plain text corpus. Thus, these models can partially alleviate the shortage of large annotated corpora, which are essential for supervised machine learning algorithms.

Without a doubt, BERT, which stands for Bidirectional Encoder Representations from Transformers, is the most popular contextualized language model due to its excellent results in many NLP applications [21]. Transformers are based on attention mechanism [20], which attempts to represent each word in a sentence based on the most relevant tokens for that word. Attention mechanisms present two major advantages compared with Recurrent Neural Networks (RNNs): first, these mechanisms can handle long-term dependencies between any two tokens in a sentence, and second, they can enable the parallelization of training.

The basic idea of BERT is that the model is trained to predict words from their contexts in an unsupervised way. This prediction only requires a large collection of texts and some strategy to mask those words to be predicted. This strategy is known as , Masked Language Modeling (MLM). First, we tokenize the texts by using the BertTokenizer class from Transformers library (provided by Hugging Face https://huggingface.co/), which offers implementations and pre-trained model weights for the most popular transformers. This class has its own vocabulary with the mappings between words and their identifiers so it is not necessary to train a tokenizer on the RareDis corpus. Each sentence is tokenized and special tokens, such as CLS and SEP, are added at the beginning and at the end of each tokenized sequence, respectively. The tokens are padded or truncated based on the maximum length (512 tokens) that the BERT-base model can handle. For each token, this class also creates a position embedding that encodes the absolute position of the token in the input sequence. It is also necessary to create an attention mask in order to distinguish which tokens correspond to real words and which ones are padding tokens. Thus, the attention mask is composed of ones (indicating non-padding entries) and zeros (indicating padding entries). The input for BERT is the masked sequence and the sum of the token and position embeddings. Then, BERT should output a vector representation for each token.

**Fig. 4:**
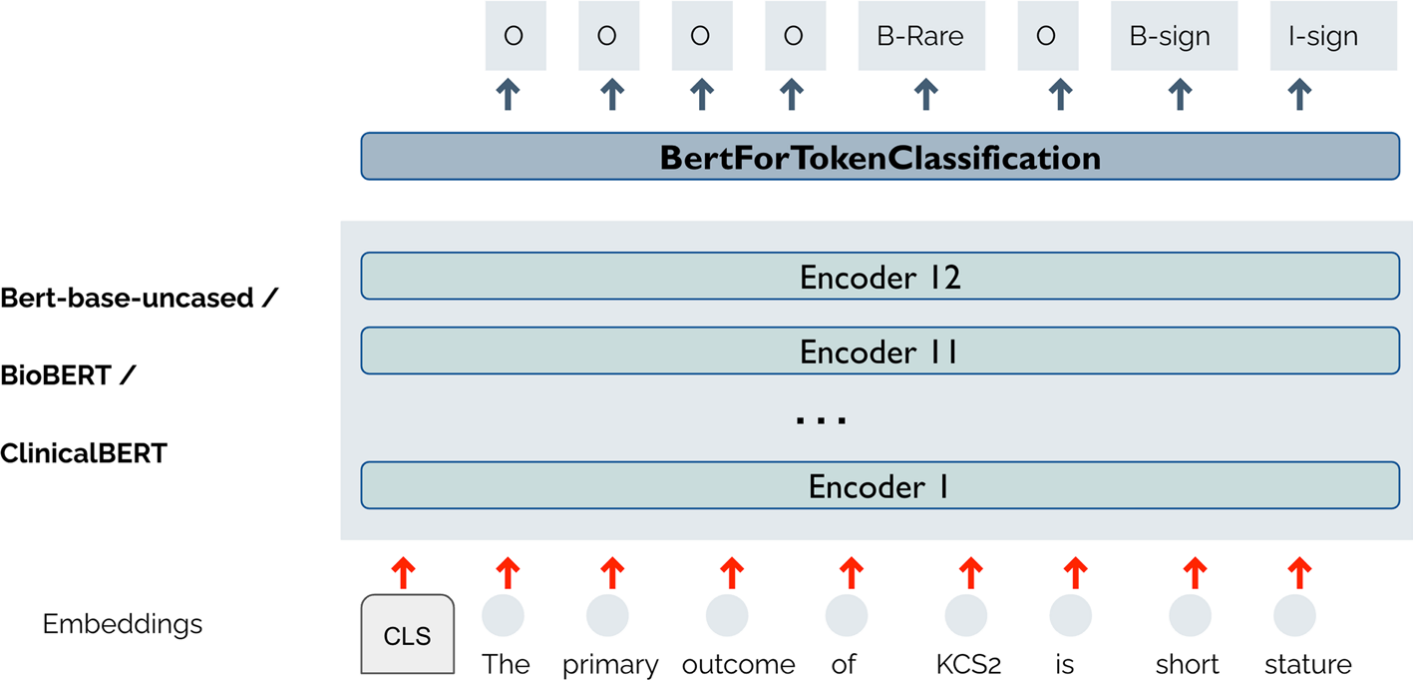
BERT-based method. This figure shows the architecture for the three BERT-based models

The architecture of BERT (which consists of 12 encoder layers for the BERT-base version) can be extended with more layers capable to solve a specific NLP task. This process is known as fine-tuning. To fine-tuning the base model (see Fig. 4), we use the “BertForTokenClassification” class from Transformers library. This class implements a token-level classifier on top of the BERT model. The token-level classifier is a linear layer that takes as input the last hidden state of the sequence and makes predictions at the token level, rather than the sequence level. Figure 4 shows the output produced by this fine-tuning model for the input sequence “The primary outcome of KCS2 is short stature”.

The BertForTokenClassification class allows to load different pre-trained models as its base architecture. In this work, we explore the following base architectures:

- Bert-base-uncased version of the original BERT proposed in [21]. This version is a stack of 12 encoders, each having 12 attention heads. For each token of the input sentence, the output layer provides an embedding of dimension 768 for this token. The total number of parameters is 110 million. The model was trained using two corpora: BookCorpus with around 800 million words and English Wikipedia with around 2500 million words.
- BioBERT [17], whose weights were initialized using the BERT weights, and then, the model was pre-trained on two biomedical corpora: PubMed abstracts (4500 million words) and PMC full-text articles (13,500 million words).
- ClinicalBERT [41] was trained with more than 2 million clinical notes from the MIMIC-III v1.4 database [60]. Its weights were initialized using the BioBERT weights.

## Results

In this section, the results obtained from the different methods are presented. We evaluate them at entity level to know how well our models predict the whole entities (for example, “ACDY5-related dyskinesia”). As complementary information, we also assess our approaches at token level. This evaluation may give us some clues as to why some entities are more difficult to recognize and what kind of tokens are more challenging for the task.

All our methods output a BIO tag for each token in the input sequence. These predicted BIO tags can be easily compared to the actual tags in the test dataset by using the sklearn library, which provides us the results at token level. That is, it calculates the scores for each label: O, B-Disease, I-Disease, B-RareDisease, I-RareDisease, B-Sign, I-Sign, B-Symptom, and I-Symptom. To evaluate the methods at entity level, we use the seqeval library [61].

NER approaches are typically evaluated in terms of recall, precision, and F1, which are calculated for each entity type or token type (BIO tags). Recall provides us how many of the predicted entities (or the predicted tokens) are correct. It can be defined as the ratio between the correctly predicted mentions for a given entity type (or token type) and the actual number of mentions of this entity type (or token type) in the test dataset. To obtain, for example, the recall for rare diseases, we have to divide the total number of rare diseases proposed by a model by the total number of rare diseases present in the test dataset. A rare disease mention is correctly identified only if all its tokens have been correctly classified with its corresponding BIO tags. Precision tells us how precise is the model. It can be defined as the ratio between the correctly predicted mentions for a given entity type (or token type) and the total number of predicted mentions by the model for this entity type (or token type). Finally, F1 is the harmonic average of precision and recall, which is a useful metric for unbalanced datasets [62]:

$$\begin{aligned} F1 = \frac{2*Precision*Recall}{Precision+Recall} \end{aligned}$$

As our task is a multi-classification problem, we also calculate micro and macro average scores. In macro averaging, metrics are calculated independently for each entity type (or for each token type), and then, we calculate the unweighted mean of these metrics. For example, the macro-average precision will be the unweighted mean of all precision scores for the entity types (or for the token types). We also compute the weighted macro-averages, in which each entity type (or token type) is weighted by the relative number of its instances in the dataset. In micro averaging, true positives, false positives, and false negatives are computed jointly for all entity types (or for all token types), and then, the metrics are calculated. In macro averaging, all classes are treated equally, while, in micro averaging, the classes with more instances will have more impact on the final performance. As our problem has a large class imbalance (see Table 1), we use micro-average scores to provide an overall comparison of all the approaches proposed in this paper.

We start by presenting the micro-average F1 of the approaches (Table 2). We can clearly see that the BERT-based models outperform all the other models. Clearly, the deep contextualized vectors from the BERT-based models provide a better representation for the input texts than those provided by CRF or the pre-trained word embeddings used in BiLSTM. BioBERT obtains better results than BERT and ClinicalBERT. This may happen because this was trained on biomedical scientific articles, whose narrative is similar to that used in the NORD database for describing rare diseases. Regarding the other approaches, although BiLSTM was extended with a CRF layer as the output layer, this architecture does not obtain better results than a simple CRF. A possible reason could be that this deep learning technique requires a larger number of training examples for learning. We now present the results of each approach below.

**Table 2:** Comparison of the methods.

Approach	F1
CRF	0.6487
BiLSTM (Wiki-PubMed-PMC)	0.4326
BiLSTM+CRF (Wiki-PubMed-PMC)	0.5805
BERT	0.6710
BioBERT	**0.6954**
ClinicalBERT	0.6810

Best micro F1 is in bold

### CRF (baseline)

Table 3 shows results achieved by CRF at entity level. CRF achieves a micro-average F1 of 64.8% and a macro-average F1 of 61.9%. As entity types are unbalanced (see Table 1), we also consider the macro-weighted-average F1, which is of 63.8%.

**Table 3:** Entity-level results of CRF

Label	Precision	Recall	F1	Support
DISEASE	0.6991	0.4912	0.5770	454
RAREDISEASE	0.8332	0.8164	0.8247	1095
SIGN	0.5313	0.3987	0.4556	958
SYMPTOM	0.7778	0.5185	0.6222	54
Micro-avg	0.7112	0.5963	0.6487	2561
Macro-avg	0.7103	0.5562	0.6199	2561
Macro-weighted	0.6953	0.5963	0.6384	2561

The best results are obtained for rare disease entity type (F1=82.4%), which is the second entity type with the largest number of instances, 5228, in the corpus (see Table 1.. On the contrary, sign entity type shows the lowest F1 (45.5%) value, even though it is the entity type with the largest number of instances, 5230 (see Table 1). Both entity types, rare diseases and signs, have a very close number of instances. This may happen because the sign mentions are usually nominal phrases (for example, “malformations of the nipples”, see Fig. 1c), unlike disease, rare disease or symptom names, which are usually a combination of few technical terms (for example, “chronic arthritis” or “ADCY5-related dyskinesia”, see Fig.  1). Token-level results are shown in Table 4. As expected, these results coincide with the results for entity-level. Its “Support” column shows the number of instances for each type of token. The number of internal tokens (I-) for diseases or rare diseases is slightly higher than the number of its initial tokens (B-), while the number of internal tokens for signs doubles the number of its initial tokens. In addition, many sign mentions are discontinuous entities, that is, they present gaps in their description. The sentence shown in Fig. 1c contains two signs: “malformations of the nipples” and “malformations of the abdominal wall”, being the last one a discontinuous mention. Another possible reason is that many signs can be also considered as diseases (see Fig. 1a). CRF and the other models proposed in this study only provide a label per token. That is, they do not address the task of overlapped entities (see Fig. 1b1). The low performance for signs can be explained by all these reasons.

**Table 4:** Token-level results of CRF

Label	Precision	Recall	F1	Support
B-DISEASE	0.7116	0.5124	0.5958	454
I-DISEASE	0.7133	0.5225	0.6032	400
B-RAREDISEASE	0.8464	0.8369	0.8416	1095
I-RAREDISEASE	0.8681	0.8261	0.8466	1179
B-SYMPTOM	0.8286	0.5800	0.6824	54
I-SYMPTOM	0.6429	0.2250	0.3333	80
B-SIGN	0.5883	0.4894	0.5343	958
I-SIGN	0.5591	0.3991	0.4658	2215
Micro-avg	0.7112	0.5818	0.6400	6243
Macro-avg	0.7198	0.5489	0.6129	6243
Macro-weighted	0.6945	0.5818	0.6292	6243

Both signs and symptoms are clinical manifestations of diseases. A sign is an objective evidence, while a symptom is a subjective experience that can only be identified by the patient. However, contrary to the low results for signs, CRF provides the second-best F1 for symptom type (F1=62.2%), even though its number of instances, 397, is the lowest in the corpus. (see Table 1). A manual review of symptoms and signs mentions in the training dataset shows that most symptoms are described by technical terms (for example, “headache”), while signs usually have lay descriptions (for example, “dark circles under eyes”). It would be necessary to increase the number of symptoms in the RareDis corpus to study whether the difference between the results of both types of entities is maintained.

### BiLSTM

All the BiLSTM models (see Table 5 provide significantly lower results than CRF (see Table 3). The decrease in micro-average F1 is more than 20% and 24% in macro-average F1. This may indicate that the training data is too small for using deep learning. As happened with CRF, BiLSTM obtains the best results for rare diseases and worst ones for signs. The results at token-level (see Table 6) are coherent with the results at entity level.

**Table 5:** Entity-level results of BiLSTM models.

Label	Precision	Recall	F1	Support
**Random initialization**				
DISEASE	0.4387	0.2913	0.3502	454
RAREDISEASE	0.4592	0.4712	0.4651	1095
SIGN	**0.328**8	0.3224	0.3256	958
SYMPTOM	0.0000	0.0000	0.0000	54
Micro-avg	0.3668	0.3742	0.3705	2561
Macro-avg	0.2454	0.2170	0.2282	2561
Macro-weighted	0.3946	0.3742	0.3820	2561
**Google news**				
DISEASE	0.4432	0.3071	0.3628	454
RAREDISEASE	0.4796	0.4971	0.4882	1095
SIGN	0.3166	0.3419	0.3287	958
SYMPTOM	0.4571	0.3200	0.3765	54
Micro-avg	0.3724	0.4020	0.3866	2561
Macro-avg	0.3393	0.2932	0.3112	2561
Macro-weighted	0.4084	0.4020	0.4028	2561
**Glove**				
DISEASE	0.4246	0.3622	0.3909	454
RAREDISEASE	0.5194	**0.5529**	0.5356	1095
SIGN	0.3114	**0.3971**	**0.3491**	958
SYMPTOM	**0.6154**	**0.4800**	** 0.5393**	54
Micro-avg	0.3850	**0.4596**	0.4190	2561
Macro-avg	0.3742	**0.3584**	0.3630	2561
Macro-weighted	0.4236	**0.4596**	0.4387	2561
**Wiki-pubmed-PMC**				
DISEASE	**0.5794**	**0.4339**	**0.4962**	454
RAREDISEASE	**0.5378**	0.5388	**0.5383**	1095
SIGN	0.3167	0.3570	0.3356	958
SYMPTOM	0.5946	0.4074	0.4835	54
Micro-avg	**0.4170**	0.4494	**0.4326**	2561
Macro-avg	**0.4057**	0.3474	**0.3707**	2561
Macro-weighted	**0.4637**	0.4494	**0.4539**	2561

Best micro and macro scores are in bold. Best scores for each entity type are also in bold

**Table 6:** Token-level results of BiLSTM.

Label	Precision	Recall	F1	Support
**Random initialization**				
B-DISEASE	0.6105	0.3102	0.4113	454
I-DISEASE	0.6447	0.3660	0.4669	400
B-RAREDISEASE	0.6232	0.5804	0.6010	1095
I-RAREDISEASE	0.7812	0.6631	0.7174	1179
B-SYMPTOM	0.0000	0.0000	0.0000	54
I-SYMPTOM	0.0000	0.0000	0.0000	80
B-SIGN	0.5930	0.3311	0.4249	958
I-SIGN	0.5924	0.4323	0.4999	2215
Micro-avg	0.6403	0.4633	0.5376	6243
Macro-avg	0.4806	0.3354	0.3902	6243
Macro-weighted	0.6227	0.4633	0.5271	6243
**Google news**				
B-DISEASE	0.6301	0.3690	0.4654	454
I-DISEASE	0.6807	0.3256	0.4405	400
B-RAREDISEASE	0.6729	0.6392	0.6556	1095
I-RAREDISEASE	0.8259	0.6375	0.7196	1179
B-SYMPTOM	0.6452	0.4082	0.5000	54
I-SYMPTOM	0.5000	0.0263	0.0500	80
B-SIGN	0.5980	0.4178	0.4919	958
I-SIGN	0.6203	0.4477	0.5200	2215
Micro-avg	0.6685	0.4906	0.5659	6243
Macro-avg	0.6466	0.4089	0.4804	6243
Macro-weighted	0.6640	0.4906	0.5593	6243
**Glove**				
B-DISEASE	0.6230	0.4198	0.5016	454
I-DISEASE	0.6320	0.4553	0.5293	400
B-RAREDISEASE	0.6838	0.6765	0.6801	1095
I-RAREDISEASE	0.8321	0.6702	0.7424	1179
B-SYMPTOM	0.6562	0.4286	0.5185	54
I-SYMPTOM	0.6667	0.1053	0.1818	80
B-SIGN	0.5937	0.5354	0.5630	958
I-SIGN	0.5994	0.5454	0.5711	2215
Micro-avg	0.6544	**0.5683**	**0.6083**	6243
Macro-avg	0.6609	**0.4796**	0.5360	6243
Macro-weighted avg	0.6568	**0.5683**	**0.6059**	6243
**Wiki-pubmed-PMC**				
B-DISEASE	0.7600	0.4718	0.5822	454
I-DISEASE	0.7546	0.5150	0.6122	400
B-RAREDISEASE	0.7163	0.6636	0.6889	1095
I-RAREDISEASE	0.8489	0.6480	0.7350	1179
B-SYMPTOM	0.6765	0.4600	0.5476	54
I-SYMPTOM	1.0000	0.0750	0.1395	80
B-SIGN	0.5318	0.5106	0.5210	958
I-SIGN	0.5807	0.4614	0.5142	2215
Micro-avg	**0.6687**	0.5369	0.5956	6243
Macro-avg	**0.7336**	0.4757	**0.5426**	6243
Macro-weighted avg	**0.6784**	0.5369	0.5934	6243

Best micro and macro scores are in bold

Regarding the effect of pre-trained word embeddings to initialize the network, the BiLSTM with Wiki-Pubmed-PMC provides the best overall results. It also obtains the best results for rare diseases and diseases. This may be because these word embeddings were trained on biomedical texts. BiLSTM with Glove achieves a slightly better F1 for signs than BiLTM with Wiki-Pubmed-PMC. However, BiLSTM with Glove achieves an improvement of almost 6% of F1 for symptoms over BiLSTM with Wiki-Pubmed-PMC. Although Glove word embeddings were not trained on biomedical texts, they obtain very close results to those obtained with Wiki-Pubmed-PMC. This may be because Glove has the biggest vocabulary size. On the other hand, random initialization shows the worst results. In fact, the model trained with random word vectors was not able to detect any symptom.

### BiLSTM-CRF

Table 7 shows the results obtained by the BiLSTM-CRF. In all the BiLSTM-CRF models, the CRF layer helps outperform the same models without using CRF, with improvements around 10–15% over the BiLSTM overall scores. All BiLSTM-CRF models achieve higher average recall scores than CRF, while their average precision scores are negatively affected. Thus, BiLSTM-CRF models still provide lower overall results than the baseline based on CRF, with a decrease of 6% in micro-average F1.

**Table 7:** Entity-level results of BiLSTM-CRF models.

Label	Precision	Recall	F1	Support
**Random initialization**				
DISEASE	0.5414	0.3780	0.4451	454
RAREDISEASE	0.6540	0.7144	0.6829	1095
SIGN	0.4892	0.4391	0.4628	958
SYMPTOM	**0.8529**	**0.5800**	**0.6905**	54
Micro-avg	0.5421	0.5494	0.5457	2561
Macro-avg	**0.5075**	0.4223	0.4563	2561
Macro-weighted	0.5748	0.5494	0.5582	2561
Google news				
DISEASE	0.5597	0.4304	0.4866	454
RAREDISEASE	0.6482	0.7548	0.6975	1095
SIGN	**0.5327**	0.4166	0.4675	958
SYMPTOM	0.6667	0.5600	0.6087	54
Micro-avg	0.5556	0.5654	0.5604	2561
Macro-avg	0.4815	0.4324	0.4521	2561
Macro-weighted	0.5887	0.5654	0.5711	2561
**Glove**				
DISEASE	0.4720	**0.5092**	0.4899	454
RAREDISEASE	**0.7226**	0.7240	**0.7233**	1095
SIGN	0.5068	**0.4606**	**0.4826**	958
SYMPTOM	0.5385	0.5600	0.5490	54
micro-avg	0.5489	0.5821	0.5650	2561
Macro-avg	0.4480	0.4508	0.4490	2561
Macro-weighted	0.5937	0.5821	0.5874	2561
**Wiki-pubmed-PMC**				
DISEASE	**0.7208**	0.4890	**0.5827**	454
RAREDISEASE	0.6339	**0.7890**	0.7030	1095
SIGN	0.4994	0.4562	0.4768	958
SYMPTOM	0.6739	0.5741	0.6200	54
Micro-avg	**0.5564**	**0.6068**	**0.5805**	2561
Macro-avg	0.5056	**0.4617**	**0.4765**	2561
Macro-weighted	**0.5998**	**0.6068**	**0.5953**	2561

Best micro and macro scores are in bold. Best scores for each entity type are also in bold

Regarding the pre-trained word embeddings, Wiki-Pubmed-PMC and Glove word embeddings provide better performance than using random initialization or GoogleNews word embeddings. BiLSTM-CRF with Glove provides the best results for rare diseases and signs, while Wiki-Pubmed-PMC provides the best F1 for diseases. Entity-level and token-level (see Table 8) results show the same behavior. The model trained with Wiki-Pubmed-PMC or Glove word embeddings achieve the best F1 scores for all token types, except for the B-Symptom and I-Symptom. For these tokens, the best F1 scores are provided by the model trained with random initialization. However, due to the lowest number of instances of this entity type, it is very difficult to give an explanation. It would be necessary to increase its number of instances to know the real behavior of the model for this entity type.

**Table 8:** Token-level results of BiLSTM+CRF models.

Label	Precision	Recall	F1	Support
**Random initialization**				
B-DISEASE	0.5714	0.3957	0.4676	454
I-DISEASE	0.5649	0.4640	0.5095	400
B-RAREDISEASE	0.6858	0.7490	0.7160	1095
I-RAREDISEASE	0.7703	0.7710	0.7707	1179
B-SYMPTOM	0.9375	0.6122	0.7407	54
I-SYMPTOM	0.8333	0.2632	0.4000	80
B-SIGN	0.6029	0.5616	0.5816	958
I-SIGN	0.6112	0.5669	0.5882	2215
Micro-avg	0.6521	0.6118	0.6313	6243
Macro-avg	0.6972	0.5480	0.5968	6243
Macro-weighted	0.6499	0.6118	0.6270	6243
**Google news**				
B-DISEASE	0.6123	0.4519	0.5200	454
I-DISEASE	0.5953	0.5130	0.5511	400
B-RAREDISEASE	0.6913	0.7990	0.7412	1095
I-RAREDISEASE	0.7727	0.8117	0.7917	1179
B-SYMPTOM	0.8108	0.6122	0.6977	54
I-SYMPTOM	0.6818	0.1974	0.3061	80
B-SIGN	0.6624	0.5308	0.5894	958
I-SIGN	0.7074	0.5236	0.6018	2215
Micro-avg	**0.7022**	0.6103	0.6530	6243
Macro-avg	**0.6918**	0.5549	0.5999	6243
Macro-weighted	**0.6992**	0.6103	0.6450	6243
**Glove**				
B-DISEASE	0.5219	0.5428	0.5321	454
I-DISEASE	0.4875	0.6167	0.5445	400
B-RAREDISEASE	0.7792	0.7510	0.7649	1095
I-RAREDISEASE	0.8009	0.8037	0.8023	1179
B-SYMPTOM	0.6739	0.6327	0.6526	54
I-SYMPTOM	0.4878	0.2632	0.3419	80
B-SIGN	0.6372	0.5753	0.6047	958
I-SIGN	0.6566	0.5730	0.6120	2215
Micro-avg	0.6789	**0.6390**	0.6583	6243
Macro-avg	0.6306	**0.5948**	0.6069	6243
Macro-weighted	0.6798	0.6390	**0.6572**	6243
**Wiki-pubmed-PMC**				
B-DISEASE	0.7616	0.5192	0.6174	454
I-DISEASE	0.7789	0.5550	0.6482	400
B-RAREDISEASE	0.6617	0.8295	0.7361	1095
I-RAREDISEASE	0.7694	0.8346	0.8007	1179
B-SYMPTOM	0.7273	0.6400	0.6809	54
I-SYMPTOM	0.6296	0.2125	0.3178	80
B-SIGN	0.5919	0.6015	0.5967	958
I-SIGN	0.5929	0.5589	0.5754	2215
Micro-avg	0.6621	0.6561	**0.6591**	6243
Macro-avg	0.6892	0.5939	**0.6216**	6243
Macro-weighted	0.6634	**0.6561**	0.6535	6243

Best micro and macro scores are in bold

As mentioned previously, BiLSTM fails to beat the baseline, not even when it includes a CRF classifier as its last layer. This may be because the training data size is not enough to train a deep learning model, while a CRF classifier trained with a simple feature set can deal with the task.

### BERT-based models

We have explored the use of three different deep contextualized word representations, all of them based on BERT (see Table 9). Unlike the BiLSTM models, these BERT-based models exceed the baseline results provided by a simple CRF classifier.

**Table 9:** Entity-level results of the BERT-based models.

Label	Precision	Recall	F1	Support
BERT base				
DISEASE	0.5197	0.6101	0.5613	454
RAREDISEASE	0.8008	**0.8667**	0.8325	1095
SIGN	0.5079	**0.6033**	0.5515	958
SYMPTOM	0.5469	0.6481	**0.5932**	54
Micro avg	0.6298	**0.7181**	0.6710	2561
Macro avg	0.5938	0.6821	0.6346	2561
Macro-weighted	0.6361	**0.7181**	0.6743	2561
**BioBERT**				
DISEASE	0.5607	**0.6608**	0.6067	454
RAREDISEASE	**0.8522**	0.8530	**0.8526**	1095
SIGN	**0.5574**	0.5877	**0.5722**	958
SYMPTOM	0.5143	0.6667	0.5806	54
Micro avg	**0.6761**	0.7157	**0.6954**	2561
Macro avg	0.6212	**0.6920**	0.6530	2561
Macro-weighted	**0.6831**	0.7157	**0.6984**	2561
BioClinical BERT				
DISEASE	**0.5788**	0.6388	**0.6073**	454
RAREDISEASE	0.8167	0.8584	0.8370	1095
SIGN	0.5296	0.5501	0.5397	958
SYMPTOM	**0.6066**	**0.6852**	0.6435	54
Micro avg	0.6625	0.7005	0.6810	2561
Macro avg	**0.6329**	0.6831	**0.6569**	2561
Macro-weighted	0.6627	0.7005	0.6810	2561

Best micro and macro scores are in bold. Best scores por each entity type are also in bold

BioBERT achieves the best micro-average and macro-weighted average F1, while the best macro-average F1 is provided by ClinicalBERT. In general, BioBERT and ClinicalBERT show very close results. As happened with the previous models, rare diseases show the best results, followed by diseases. BioBERT obtains the best F1 for rare diseases and for signs, while ClinicalBERT BERT provides the best results for diseases and symptoms. As expected, the BERT base model, which was trained on BookCorpus and English Wikipedia, obtains lower results than BioBERT and ClinicalBERT.

Regarding the results at the token-level (see Table 10), BioBERT achieves the best F1 scores for all token types, except for B-Symptom and I-Symptom tokens. In these tokens, the best model is ClinicalBERT. Comparing to the previous approaches, all BERT-based models achieve significant improvements on recall. For example, BioBERT largely outperforms CRF, with an increase of 17 points in recall for diseases (see Table 3). The BERT-base also shows significant improvement on recall for rare diseases and signs, with differences of 5 and 14 points, respectively, comparing to the previous best model BiLSTM-CRF. Similarly, ClinicalBert has an improvement of 10 points for recall over recall provided by BiLSTM-CRF model. This significant improvement on recall compared to the previous method may be due to

**Table 10:** Token-level results of the BERT-based models.

Label	Precision	Recall	F1	Support
**BERT base**				
B-DISEASE	0.6012	0.6637	0.6309	454
I-DISEASE	0.5186	0.5884	0.5513	400
B-RAREDISEASE	0.8451	0.9003	0.8718	1095
I-RAREDISEASE	0.8704	0.9024	0.8861	1179
B-SYMPTOM	0.6607	0.7400	0.6981	54
I-SYMPTOM	0.6000	0.4918	0.5405	80
B-SIGN	0.6514	0.7073	0.6782	958
I-SIGN	0.6725	0.7099	0.6907	2215
Micro avg	0.7353	0.7794	0.7567	6243
Macro avg	0.6775	0.7130	0.6935	6243
Macro-weighted avg	0.7379	0.7794	0.7579	6243
**BioBERT**				
B-DISEASE	0.6356	0.7088	0.6702	454
I-DISEASE	0.5716	0.6964	0.6279	400
B-RAREDISEASE	0.8825	0.8816	0.8821	1095
I-RAREDISEASE	0.9142	0.8927	0.9033	1179
B-SYMPTOM	0.6349	0.8000	0.7080	54
I-SYMPTOM	0.5538	0.5538	0.5538	80
B-SIGN	0.7238	0.7049	0.7142	958
I-SIGN	0.7330	0.6978	0.7150	2215
Micro avg	0.7830	**0.7855**	**0.7842**	6243
Macro avg	0.7062	**0.7420**	0.7218	6243
Macro-weighted avg	**0.7890**	**0.7855**	**0.7863**	6243
**ClinicalBERT**				
B-DISEASE	0.6503	0.6885	0.6689	454
I-DISEASE	0.5969	0.6557	0.6249	400
B-RAREDISEASE	0.8614	0.8807	0.8710	1095
I-RAREDISEASE	0.8829	0.9076	0.8951	1179
B-SYMPTOM	0.7547	0.8000	0.7767	54
I-SYMPTOM	0.7158	0.5231	0.6044	80
B-SIGN	0.6996	0.6961	0.6979	958
I-SIGN	0.7575	0.6220	0.6831	2215
Micro avg	**0.7881**	0.7609	0.7742	6243
Macro avg	**0.7399**	0.7217	**0.7277**	6243
Macro-weighted avg	0.7873	0.7609	0.6243	11,909

Best micro and macro scores are in bold

Given text, WordPiece first pre-tokenizes the text into words (by splitting on punctuation and whitespaces) and then tokenizes each word into subword units, called wordpieces

## Discussion

Although rare diseases have a very low prevalence in the population, approximately 6% of the world’s population suffer a rare disease. This number is continually growing as five new rare diseases are discovered each week [63].

In this paper, we study several methods for recognizing rare diseases and their clinical manifestations. We propose a CRF baseline system using linguistic features. Second, we implement multiple BiLSTMs, testing different classifiers at the output layer such as softmax or CRF, as well as exploring different strategies to initialize their input vectors, such as random initialization and three pre-trained word embedding models, one of them was trained on biomedical texts. Moreover, we explore three implementations of BERT, which differ between them by the type of texts used to pre-train the model. The RareDis corpus is used to train the models and evaluate them. The experiments show that BioBERT obtains the best micro and macro-weighted-average F1, with improvements around 5% over the baseline (CRF) results. BiLSTM does not even outperform the baseline in terms of F1.

Regarding the entity types, the best model, BioBERT, provides the highest F1 (85.2%) for rare diseases, followed by diseases with a F1 of 60.7%. Rare disease names are usually more complex than disease names since many rare disease names often contain disease names (for example, ”central diabetes insipidus”). Therefore, the difference between these results may be that the number of rare diseases mentions is twice the number of diseases mentions in the dataset (see Table 1). The other entity types, sign and symptom, do not outperform 60% in F1. One possible reason for the low results to recognize symptoms (F1=58%) may be the insufficient number of training instances for this entity type (see Table 1). On the other hand, although sign is the majority class (see Table 1), this shows the lowest F1 (57.2%). In this case, the most probable reason is that many signs are usually described by complex noun phrases that often involve the use of overlapped, nested and discontinuous entities.

As mentioned before, the BERT-base models provide significant improvements on recall scores for all the entity types and all the token types, compared to the previous approaches. This large improvement may be explained by the wordpiece tokenization used in BERT, while the previous approaches used word-based tokenizers to process the input texts. The wordpiece tokenizer first splits the text into tokens, and then the rare words are broken into smaller meaningful words (named wordpieces) [64]. For example, as “chronic” is a very common word, it is not split into smaller subwords. In contrast, “arthritis” can be considered a rare word because it is less common than “chronic”. For this reason, the tokenizer breaks it into three wordpieces: “art”, “##hr”, “##itis”. Wordpiece tokenization has multiples advantages. First, it provides a representation for unknown words by splitting them into known smaller tokens. Moreover, the model is able to learn meaningful representations with a reasonable vocabulary size. These advantages have been also mentioned in several previous works [65–67].

Most previous work has focused on recognizing disease names. So far, a model based on transformers [18] has achieved the state-of-the-art results with an F1 of 89.92% on the NCBI corpus, and 93.82% on the CDR corpus. Our results are not directly comparable to previous work, because this is the first work that addresses the detection of rare diseases and their clinical manifestations from the RareDis corpus. Even so, we can see that our model based on BioBERT provides an F1 of 85.25% for the recognizing of rare diseases, which is close to the state-of-the-art performance on the NCBI corpus (F1=89.92%)). We should note that the NCBI corpus contains 6982 disease mentions, while our RareDis corpus only has 5228 rare disease mentions (3608 instances in the training subset). That is, it is reasonable to think that if the RareDis corpus had a similar number of instances to the NCBI corpus, our model could have achieved close results to the state-of-the-art results on the NCBI corpus [18]. Similarly, the size of the CDR corpus is also greater than the RareDis corpus. Moreover, until now, only one study addressed the task of recognizing rare diseases and their disabilities from texts, by using a BiLSTM with a CRF layer. This approach achieved an F1 of 70.1% for rare diseases on the RDD corpus [40]. Therefore, our approach using BioBERT obtains a better performance.

As future work, we plan to extend the size of the RareDis corpus by including MedLine abstracts and clinical cases of rare diseases. This will increase the number of instances for all entity types, especially the number of instances for symptoms. It could have a significant positive effect on the results, especially those achieved by the deep learning models. We also plan to extend the corpus with texts written in other languages than English. Thus, we will study the behavior of our models on other type of texts.

We will also address some unsolved problems in NER such as the recognition of nested, overlapped and discontinuous entities, which could improve the results for signs. Regarding the models, we will study on fine-tuning the BERT-based models by adding different techniques, such as a simple CRF or more complex architectures. Furthermore, we plan to address the task of relation extraction on the RareDis corpus.

## Data Availability

The dataset supporting the conclusions of the current study is available in the NLP4RARE-CM-UC3M repository, https://github.com/isegura/NLP4RARE-CM-UC3M. Our source code is also publicly available to enable the reproducibility of our experiments at https://github.com/cadovid/nlp4rare

## Footnotes

1 https://rarediseases.org/

2 https://github.com/isegura/NLP4RARE-CM-UC3M
